# Benefits of Post-Graduate Courses

**Published:** 1905-08-15

**Authors:** A. C. Runyan

**Affiliations:** South Haven


### BENEFITS OF POST-GRADUATE COURSES.

BY A. C. RUNYAN, SOUTH HAVEN.

Read before the Southwestern Michigan Dental Society, April 12, 1905.

The benefits of post-graduate work is rather a large subject, and embraces so many points of interest that I find it difficult to incorporate them all in one short article. However, I will mention some of them and suggest others that may lead to discussion, and in that way lead others, who are more capable of handling the matter than I am, to talk on the subject. There are several present at this meeting who have taken post-graduate work at the university and I should be pleased indeed to hear from them, as I desire to hear the matter discussed from all standpoints. As for myself, I can only speak of the benefits which I have experienced.

What I consider one of the principle benefits is that it gives the practitioner the benefits of the experience of a specialist along some particular line of practice or research, who imparts not only the knowledge he has acquired but a part of his personality and enthusiasm.

While our journals and societies keep us posted and thoroughly abreast with the progress of our profession, and we may, by hard work and many failures, work out for ourselves the required results, it is far more satisfactory, and I believe cheaper, from a financial standpoint, to get your inspiration from the fountain head.

There are several reasons for this. The principal one is, that it matters not how thoroughly an operation may be described, or how minutely the "modus operandi" may be written, there are little features of technique that are entirely lost which very often may be the principal features of success.

There is another important feature in post-graduate work which must not be over-looked. You are brought into immediate contact with the most progressive spirits from all parts of the country, who have come on the same errand as yourself, so that you not only get that which you pay for in the school of instruction, but you reap the benefit of the experience of these men on many different subjects. The practitioner who will take the time and money to improve himself in some special line of work, is usually a man that no other practitioner can meet without being benefitted, professionally, at least. The inspiration imparted by a body of thoroughly interested men who have come by experience to thoroughly know what they need, will accomplish more in the two or four weeks usually given to a post-graduate course than many of us did in the same number of months in our school work.

Then there is the social feature, particularly if there are several rooming or boarding at the same place. All are benefitted by telling of the pleasant, and sometimes not so pleasant, experiences of the every day routine of office practice. This, of course, is only incidental, but they all go toward the general benefits derived. There are some who feel that they cannot spend the time and money, but if you will put it in the way of a vacation you will receive all the benefits that a vacation would give you, spend less money and return to your practice with new inspirations. You will certainly have a greater regard for your chosen profession and be better prepared to serve your patrons. Consequently you are a better man in every particular. I think that our university at Ann Arbor, has taken a wise step in providing a time for post-graduate work along special lines, where her alumni and others may come and renew their youth and gather new inspirations. By availing ourselves of this privilege we shall make progress and never grow stale, and the standard of our profession shall be elevated above the plane of commercialism and quackery. I hope that every member of this society will do all he can to encourage and sustain our university in this work, as it is done without the expectation of profit and wholly that the general practitioners of the State may receive the benefits.

### DISCUSSION.

Dr. A. S. Rowley: As old a practitioner as I am, I shall be glad to know that there is such an opportunity for me to refresh my knowledge and hope to avail myself of the universities' liberality some time. The old men need such things, as the essayist says, to keep them up-to-date and from falling into routine practice. My experience suggests that many young practitioners need it badly, as they failed to take advantage of their opportunities when in college, and are not capable of doing some of the more common place operations. After a student has practiced for a year or so, he begins to find out what he don't know and ought to have learned while he was in college, and an opportunity of this kind ought to be appreciated by him.

Dr. S. M. White: As long as we can keep patients coming our way we don't feel the need of more knowledge. I think one of the best post-graduate courses a young man can have is to attend all the dental societies he can reach. He will get inspiration from the papers and discussions, and learn skill from the clinics, and find out what other professional men are thinking about and doing. No man can profitably to himself or his patients, shut himself off from his professional brethren.

Dr. W. W. Scott: I have had a long experience in trying to keep up with the profession by reading and attending society meetings. I don't know as this has been the reason why I have held my patients, but I believe it has had much to do with it. I want to and do learn something every day. I don't know as I could leave my practice to go to school again, but I think I could learn something there that would be profitable for both, me and my patients.

DR. RIX: It has been my practice for forty-seven years to attend every dental gathering I could reach where there was a prospect of learning something. I am still young enough to take university course and may do so. I have always had associated with me in my practice young men, generally college graduates. Sometimes they have known very much more than I, and occasionally I have been able to instruct them a little. I have always made it a practice to learn all I could from every source, because I find it very useful to know quite a few things. You never know when you will be called to do something unusual. Association with members of the profession is, in every way, helpful and ought to be encouraged.

DR. E. A. HONEY: I suspect some men who are over sensitive fear that it would be an acknowledgment of inefficiency to have it known that they had gone off to school again. I think this a very erroneous idea. I recently took the post-graduate course in porcelain work at the university and feel greatly repaid for the time and money it cost me. You can't get into such work with a lot of other earnest men without receiving benefit. The benefit will usually be in proportion to the interest one takes and the earnestness with which he employs his time. One can't very well avoid getting helpful suggestions and new inspirations. This is a means of advancing our technical knowledge and skill that it seems to me we ought to encourage.

DR. C. H. WARBOYS: Like the previous speaker I took the porcelain course at the university, and like the boy with the pie, "it tasted so good that it sounds like more." We had a two-weeks dental meeting with clinics all the time. We visited and worked both day and night, and no subject was left undiscussed. Every one seemed to know something he was glad to tell, and we were all just as eager to hear of it, and approve or disapprove as the case might be. We had clinics and were not afraid to work before each other and have our work inspected by the professors or any one else. We made failures and learned valuable lessons from them as we had some one to put us right without long series of useless experimentation. I consider it an ideal method of improving one's skill and of getting new ideas and enthusiasm. Everybody ought to get into this course, the more the greater the benefit.

Dr. Hoff: I would say in reference to this course, that it is an effort, as the essayist has said, on the part of the dental faculty of the university to put at the disposal of the profession in this State the facilities used for instructing students. We hope for two things, First, that the profession will show its appreciation by availing itself of this opportunity to secure near home instructions of the highest order in technical affairs for a reasonable fee, that it has never had an opportunity to secure except by going a long distance and obtaining at great expense, and, Secondly, that this will result in inspiring practitioners of our State to contest with each other in the attainment of superior skill, so that in time we shall have all the clinicians necessary to demonstrate every new or valuable method in any of our local or state societies without sending abroad to import some one, in order that our meetings may not be failures so far as the clinics are concerned. If the profession appreciates this effort enough to warrant us in continuing it, we shall be glad to do so, although it involves much extra labor and brings no profit whatever to the university.

